# Emotions and worries during 1.5 years of the COVID-19 pandemic - how adults with and without mental health conditions coped with the crisis

**DOI:** 10.1186/s12888-024-05573-x

**Published:** 2024-02-09

**Authors:** Josefine Rothe, Greta Brückner, Melanie Ring, Veit Roessner, Nicole Wolff, Nora C. Vetter

**Affiliations:** 1 Department of Child and Adolescent Psychiatry and Psychotherapy, Technische Universität Dresden, Dresden, Germany; 2 Faculty of Natural Sciences, Department of Psychology, Medical School Berlin, Berlin, Germany

**Keywords:** Psychosocial stress, Crisis, SARS-CoV-2, Mental health, Lockdown, Coping

## Abstract

**Background:**

During the COVID 19 pandemic, there were social restrictions with severe mental stress for a long time. Most studies on mental health consequences of the pandemic focused primarily on the beginning of the pandemic. The present study on families of patients or study participants of a child and adolescent psychiatry aimed to examine long-term profiles of emotions and worries in adults with and without mental health condition (mhc) during the first 1.5 years of the COVID-19 pandemic.

**Methods:**

We surveyed emotions and worries of 128 adults with (*n* = 32) and without (*n* = 96) pre-pandemic mhc over a 1.5-year study period from spring 2020 until summer/autumn 2021. Emotions and worries were captured at four time points: [i] pre-pandemic, [ii] spring 2020 (first lockdown was implemented), [iii] December 2020 (hard lockdown at Christmas time) and [iv] summer/autumn 2021 (considerable ease of regulations); [i] pre-pandemic and [iii] December 2020 were measured retrospectively). First, we run non-parametric tests to compare emotions and worries between adults with and without pre-pandemic mhc at the four time points. Next, we conducted latent profile analysis to identify subgroups from the total sample who share similar trajectories of emotions and worries. Finally, a logistic regression analysis was run to examine whether socio-demographic and psycho-social factors were related to identified trajectories of emotions and worries.

**Results:**

Adults without pre-pandemic mhc reported a strong worsening of emotions and worries at the beginning of the pandemic and a lower worsening during the course, while adults with pre-pandemic mhc reported a constant worsening of emotions and worries. The latent profile analysis revealed three profiles of adults who show either i) an adaption, ii) no adaption or iii) a continuous high condition. With increasing age, higher perceived stress and pre-pandemic mhc, the likelihood of an adaption was increased.

**Conclusion:**

The results of the present study suggested that adults (both with and without pre-pandemic mhc) coped the crisis with different strategies and that most of them returned to their initial, pre-pandemic levels of emotions and worries when social restrictions were considerably eased or stopped.

**Supplementary Information:**

The online version contains supplementary material available at 10.1186/s12888-024-05573-x.

## Background

The Corona Virus Disease 2019 (COVID-19) pandemic with its social restrictions and consequences and the resulting negative emotions and worries has influenced mental health [1–4]. Cross-sectional and longitudinal studies examined psychological, social and emotional wellbeing as well as symptoms of mental health conditions (mhc; e.g., loneliness, depressive symptoms, anxiety) of experiencing COVID-19 related social restrictions and consequences e.g. [5–15]. Findings from the adult German and British general population showed no significant change of symptoms of mhc [12, 15, 16] or even improved mental health compared to pre-lockdown (situation before severe restriction of social contacts and scope of movement) [5]. Ahrens and colleagues [5] mentioned overall improved mental health in a German adult population-based study (as part of the longitudinal resilience assessment study, LORA) in the 8 weeks after the first lock-down (period of severe restriction of social contacts and scope of movement) compared to the pre-lock-down period which corresponded to the reduced frequency of mentioned daily hassles. On the other hand, the authors emphasized the existence of interindividual differences in vulnerability in mental health. While two subgroups improved in their mental health or at least returned to baseline level (=pre-lock-down) 8 weeks after the first lock-down, a vulnerable subgroup of adults was more affected by the COVID-19 related social restrictions and consequences in the long run. A recent British longitudinal study measuring weekly levels of depression and anxiety scores over the course of 20 weeks, found that in general, symptoms of mhc declined across the first 20 weeks following the introduction of COVID-19 related social restrictions [12]. Taking a closer look, interindividual differences became apparent: e.g., women, younger adults and people with lower levels of educational attainment showed higher levels of anxiety and depressive symptoms at the beginning of the COVID-19 related social restrictions but also ongoing and faster improvements in symptomatic levels over the course of 20 weeks. The authors also reported that levels of anxiety and depressive symptoms in adults living with at least one child (independently of other adults living in the household) rather than in adults living with other adults but no children improved significantly faster across the first 20 weeks following the introduction of COVID-19 related social restrictions. Literature also suggests different predictors for mental health during the pandemic, such as low income [5, 12, 17, 18] or female gender [8, 12, 16].

Adults with mhc seem to present a higher vulnerability towards showing negative mental health (e.g., more worries, higher levels of depression, anxiety, contamination fears and feeling lonely) due to COVID-19 related social restrictions and consequences [19–21]. For instance, Kwong and colleagues [19] showed that during the pandemic, adults with pre-pandemic mhc reported elevated frequencies of additional symptoms of mhc. In contrast, our group found a greater number of emotions worsened significantly at the beginning of the pandemic (pre-pandemic and spring 2020) for adults without as compared to those with pre-pandemic mhc [22]. We concluded that in spring 2020 adults with pre-pandemic mhc experienced fewer negative changes as social contacts are often associated with anhedonia and fear and they often live socially withdrawn independently of the COVID-19-related social restrictions and consequences (e.g. [23]). Furthermore, they might benefit from previously learned coping strategies (such as searching support, acceptance, changing perspective and problem solving) [24] and decreased external demands as well as eased daily hassles [5]. By now, there is further evidence of rather constant emotional load from the pre-pandemic period to the beginning of the pandemic and through the first year of the pandemic for adults with a pre-pandemic mhc compared to worsened emotions in pre-pandemic mentally healthy adults [25, 26]. Increasing social support [25] seems to be protective to detrimental effects of COVID-19-related stressors in both adults with and without pre-pandemic mhc and may play a key role in supporting adaptive coping behaviours [27].

In addition to the mentioned interindividual predictors for mental health during the pandemic (e.g. low income, female gender, age, educational level, living with at least one child, pre-pandemic mhc) coping styles seem to be further interindividual predictors for mental health symptoms during the pandemic [28–30]. While an emotion-diverting coping style (e.g. self-distraction and venting) as well as an avoidant coping style (e.g. denial and behavioural disengagement) are predictors for (sub)clinical stable or increasing depressive symptoms, a constructive coping style like positive reframing and acceptance is a protective factor against moderate or increasing depressive symptoms [28–30]. However, cultural factors such as uncertainty avoidance or masculinity (for more details see Hofstede’s cultural dimensions [31]) seem to moderate the relationship between coping style and depressive symptoms [32]. So far, only the study of Cheng at al. 2023 has examined the effects of cultural differences in the relationship between coping style and mental health at a national level. Yet, cultural differences such as uncertainty avoidance or masculinity not only vary between countries, but also vary within countries respectively between regions (for an overview of regional cultural differences within Europe see [33]). Identifying cultural differences at the regional level requires the regional allocation of samples, which is not captured and/or reported in most population-based samples.

**Fig. 1:**
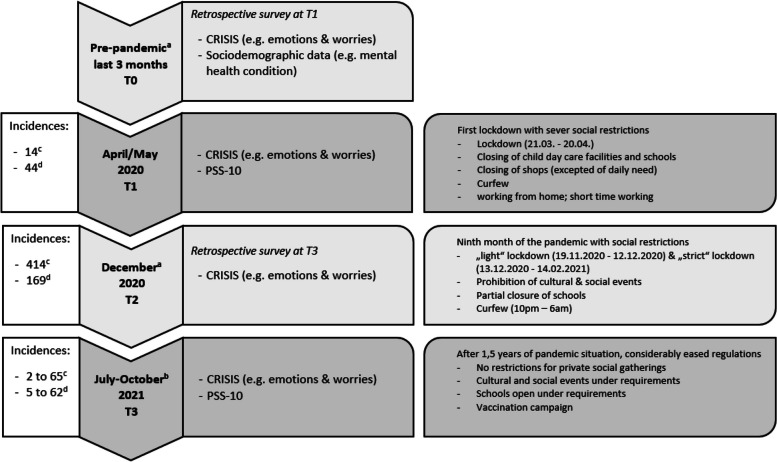
Study design including infection cases (overall cases & 7-day incidences of COVID-19) and social restrictions. Note. All reported data and regulations are from the Robert Koch Institute (RKI) and the federal state of Saxony; reported incidences are 7-day incidences of COVID-19; reported regulations are exemplary; ^a^ retrospective self-report; ^b^ mid-July until mid-October; ^c^ data of Saxony; ^d^ data of Germany

The complex, inconsistent picture drawn by the above-mentioned findings stresses the importance of evaluating the pandemic’s impact not only on pre-defined groups based on manifest variables (categorial approach) but rather on latent profiles (continuous approach). People differ in their vulnerability level leading to potentially adverse outcomes. In addition, it is important to investigate how individuals in certain regions have coped with the crisis in order to make cultural differences visible. The current study on families of current and former patients or study participants of a child and adolescent psychiatry in Saxony (a federal state in eastern Germany) aims to examine the change of emotions and worries using the *CRISIS* questionnaire during the pandemic over a 1.5-year period in adults with a pre-pandemic mhc and those without a pre-pandemic mhc. We captured 10 emotions and worries (described in the online supplementary material) at four time points. For an overview of the four time points with the related measures and the corresponding COVID-19 regulations, overall cases and 7-day incidences of COVID-19 please see Fig. 1. We hypothesised first, that in our sample of families associated with the child and adolescent psychiatry (current and former patients or previous study participants) adults with pre-pandemic mhc experienced less negative changes in emotions and worries through the COVID-19 related social restrictions and consequences than adults without pre-pandemic mhc as i) they might benefit from previously learned coping strategies [24] in response to constraints or setbacks due to their mhc as well as strained family relationships [23] and ii) social contacts in adults with mhc are often associated with anhedonia and fear and they often live socially withdrawn. Second, we assumed to identify at least two profiles for the course of emotions and worries over the 1.5-year study period, a reactive (increase at the beginning of the pandemic and back to pre-pandemic level) and a constant course of negative emotions and worries whose membership can be predicted by pre-pandemic mhc, perceived stress and female sex [5].

## Methods

### Study design and participants

Families of current and former patients of the Clinic of Child and Adolescent Psychiatry and Psychotherapy of the Universitätsklinikum Carl Gustav Carus Dresden, families of previous study participants and young adults (without children) were initially invited to take part in the survey in spring 2020 (please refer to [22] for more details). Parents were invited to report about both their child(ren) and themselves. As a follow-up survey of the study by our group [22], a total of 352 adults that consented to the inquiry in 2020 and agreed to being surveyed again were asked to complete slightly adapted online questionnaires as in 2020, the *CRISIS* [34] and the German short-version of the Perceived Stress Scale (PSS-10) [35, 36]. Responses of the follow-up survey were gathered from 16th of July to October 25th 2021.

In sum, the participants were asked to report about four time points, two of them retrospectively (see Fig. 1). In spring 2020, participants reported retrospectively about their emotions and worries in the 3 months prior to the pandemic (time point 0), as well as about their emotions and worries and their perceived stress in the last 2 weeks, i.e. during the first lock down (time point 1). In summer/autumn 2021 participants were surveyed retrospectively about their emotions and worries during a hard lockdown at Christmas time in December 2020 (time point 2) as well as about their emotions and worries and their perceived stress in the past 2 weeks (time point 3). In a previous study by our group [22] we reported only data from T0 and T1 of the initial larger sample. Details of the COVID-19 related social restrictions and consequences in Germany during the four time points are described in the online supplementary material of the present study.

A total of 128 adults (mean age 37.9 years, age range 18–56 years) took part in both surveys (spring 2020 and summer/autumn 2021). The response rate of the present study (36.4%; 128 out of 352) corresponds to the average response rate of 44.1% examined in about 1071 online surveys in education-related research [37]. Characteristics (sex and age) of the sample with complete *CRISIS*-data for all four time points (pre-pandemic, spring 2020, December 2020, summer/autumn 2021; *N* = 128) are displayed in Table 1, separated for participants with self-reported pre-pandemic mhc (*n* = 32) and without (*n* = 96). Participants with pre-pandemic mhc reported pre-pandemic mental disorders (partly several per participant) of the following categories: mood disorders (71.9%), neurotic, stress-related and somatoform disorders (25%), disorders of adult personality and behaviour (12.5%), eating disorders (9.4%), and chronic fatigue syndrome once.

**Table 1:** Sample characteristics

	**Adults with pre-pandemic mhc (*n*** **= 32)**	**Adults without pre-pandemic mhc (*n*** **= 96)**	**Test statistic**	***p*** **value**	**Effect size**
Sex, frequency (male/female)	0/32	18/78	*X*^*2*^ (1) = 6.98	.006	*φ* = .23
Age T3, *M* (SD)	37.41 (9.11)	38.70 (11.07)	*t* (126) = −.60	.55	*d* = .12
range	18–54	18–56			
	**Adults with pre-pandemic mhc** (*n* = 29)	**Adults without pre-pandemic mhc** (*n* = 88)	**Test statistic**	**p value**	**Effect size**
Perceived Stress Scale, mean t-value T1 (SD)	62.22 (14.12)	54.40 (12.76)	*t* (115) = 2.79	.006	*d* = .60
Perceived Stress Scale, mean t-value T3 (SD)	65.80 (11.98)	56.63 (13.09)	*t* (115) = 3.34	.001	*d* = .72

*mhc* mental health condition. As the PSS-10 was not answered by all included participants, the mean t-values for perceived stress are shown for a smaller subsample with complete perceived stress data

The survey was approved by the local ethics department (reference number: EK 356092018) and conducted according to the recommendations of the Helsinki Declaration. All participants provided informed consent.

### Measures

#### Socio-demographic measures

The following socio-demographic measures were captured: age, sex, education, urban (or rural) living, minors living in the same household, get social welfare, financial worries. Information’s about the scales of socio-demographic measures are described in the online supplementary material.

#### Mental health condition (mhc)

Pre-pandemic mental health condition was captured by self-reports of the participants. They were asked whether a physician has ever diagnosed a mental disorder and if so, which mental disorder.

#### CoRonavIruS health impact survey (CRISIS)

We used the CoRonavIruS Health Impact Survey (*CRISIS*) to record health and exposure status to COVID-19 as well as life changes due to COVID-19.

Daily behaviours, emotions and worries as well as media and substance use were assessed on a 5-point scale. Furthermore, exposure status to COVID-19 as well as life changes due to the pandemic (i.e. financial worries related to the COVID-19 pandemic, and changes in the quality of relationships with family and friends) were assessed. All questions of the CRISIS related to the previous 2 weeks. In addition, emotions and worries were surveyed at T1 and T3 for a longer retrospective period. For data analysis, all scales were transformed so that higher values indicate worsening of positive and negative emotions and worries.

#### Perceived stress scale (PSS-10)

The well-established Perceived Stress Scale [35], measuring whether life situations are classified as stressful on a 5-point response scale, was used to assess stress levels during the past 2 weeks. Good internal consistency (Cronbach alpha = .84) was reported for the German version of the 10-item scale (maximum score = 40) [36]. For analysis, t-scores based on norms of the Harris Poll [38] were used.

### Statistical analysis

To identify differences in sex between participants with and without pre-pandemic mhc a chi-square test was run. Independent sample t-tests were conducted to investigate the difference in age and the t-scores of perceived stress measure (PSS-10) between participants with and without pre-pandemic mhc. The 10 *CRISIS*-items regarding emotions and worries showed a good internal consistency (Cronbach alpha = .89) for pre-pandemic values within our total sample. As data on the 10 emotions and worries were measured by a 5-point scale (so they are ordinal scaled) and do not show a normal distribution, Wilcoxon signed-rank tests were used to compare emotions and worries between the four time points respectively for participants with and without pre-pandemic mhc (hypothesis 1). In addition, Mann–Whitney-U-tests were run to compare pre-pandemic emotions and worries between the groups. False discovery rate (FDR) correction [39] was applied to correct for multiple testing.

To deduce discrete latent variables that describe distinct subgroups of participants who share similar trajectories of emotions and worries, a latent profile analysis (LPA) was conducted (hypothesis 2). Four models were examined with increasing numbers of profiles: (1) a model with equal variances and covariances fixed to 0, (2) a model with varying variances and covariances fixed to 0, (3) a model with equal variances and equal covariances, and (4) a model with varying variances and varying covariances. The best fitting model was determined by an analytic hierarchy process, based on: Bayesian Information Criterion (BIC), Akaike’s Information Criterion (AIC), Approximate Weight of Evidence (AWE), Classification Likelihood Criterion (CLC), Kullback Information Criterion (KIC), according to [40]. We further inspected entropy. Entropy values ≥0.80 are associated with an assignment accuracy of 90%, which is considered an acceptable degree of separation between classes [41].

Next, we tested whether profile membership could be predicted by socio-demographic and psycho-social factors (age, sex, education, urban or rural living, have minors living in the same household, pre-pandemic mhc, get social welfare, financial worries, perceived stress; all surveyed at the beginning of the pandemic at T1) we conducted a multinomial logistic regression. We tested the continuous predictor variables a priori to verify that there was no violation of the assumption of the linearity of the logit and multicollinearity.

The tidy LPA package [42] working in the RStudio 2022.07.2 (R 4.1.2) environment was used to conduct the LPA, while SPSS 28 was used for all other analysis.

## Results

### Changes in emotions and worries from pre-pandemic values (2020) until summer/autumn 2021

Means and standard deviations for the 10 emotions and worries assessed by the *CRISIS* at the four time points as well as effect sizes of pre-pandemic group-differences (with mhc/without mhc) using Mann–Whitney-U-tests are displayed in Table 2. In half of pre-pandemic (T0) emotions and worries (enjoy activities, concentrated, negative thoughts, happy or sad, relaxed or anxious) participants with pre-pandemic mhc had worse values as compared to participants without pre-pandemic mhc.

**Table 2:** Means and standard deviations of reported emotions and worries of the *CRISIS* and effect sizes *r* of pre-pandemic group-differences using Mann–Whitney-U-tests

Item	Adults with pre-pandemic mhc (*n* = 32)				Adults without pre-pandemic mhc (*n* = 96)				pre-pandemic group-difference
	M^pre^ (SD)	M^T1^ (SD)	M^T2^ (SD)	M^T3^ (SD)	M^pre^ (SD)	M^T1^ (SD)	M^T2^ (SD)	M^T3^ (SD)	*r*
Worried	2.12 (1.16)	2.78 (.91)	3.28 (1.25)	2.44 (1.32)	1.73 (.79)	2.54 (.92)	2.81 (1.00)	1.68 (.79)	.14
enjoy activities	2.78 (1.04)	3.37 (1.16)	3.94 (1.11)	2.78 (1.21)	2.09 (.93)	2.96 (1.07)	3.31 (1.12)	2.36 (.87)	.29*
Concentrated	2.81 (1.12)	3.06 (1.01)	3.37 (1.29)	3.16 (1.37)	2.11 (1.09)	2.51 (1.07)	2.61 (1.15)	2.34 (1.13)	.27*
Lonely	1.97 (1.09)	1.97 (1.20)	2.78 (1.45)	2.16 (1.42)	1.47 (.71)	1.79 (.89)	1.82 (1.01)	1.55 (.88)	.21
negative thoughts	3.25 (1.05)	3.06 (1.01)	3.53 (.92)	3.38 (1.10)	2.49 (.95)	2.49 (1.00)	2.57 (.95)	2.29 (.96)	.31*
happy or sad	3.13 (1.04)	3.28 (1.05)	4.03 (1.06)	3.72 (1.25)	2.38 (.95)	2.74 (.97)	3.35 (.94)	2.69 (1.08)	.30*
relaxed or anxious	3.22 (.91)	3.41 (.95)	3.69 (1.03)	3.28 (1.22)	2.33 (1.05)	2.78 (1.02)	3.10 (.98)	2.34 (1.00)	.35*
fidgety or restless	2.13 (1.13)	2.16 (1.11)	2.59 (1.21)	2.19 (1.20)	1.83 (.87)	2.06 (1.01)	1.89 (1.04)	1.60 (.84)	.10
fatigued or tired	3.06 (1.22)	2.97 (1.20)	3.56 (.84)	3.34 (1.23)	2.47 (.97)	2.44 (1.06)	2.91 (1.22)	2.32 (1.11)	.21
irritable or easy angered	2.41 (.95)	2.59 (1.10)	2.81 (1.23)	2.81 (1.15)	1.98 (.85)	2.32 (1.05)	2.54 (1.09)	2.09 (.99)	.19

* results are significant after FDR correction

Over the total 1.5-year study period the Wilcoxon signed-rank tests indicated the strongest effect sizes on changes in emotions and worries about “be worried”, “enjoy activities” and “be happy or sad”, both in participants with and without pre-pandemic mhc (for effect sizes of these three items see Table 3, for all results of the series of Wilcoxon signed-rank tests see Tables A1 and A2 in online supplemental material). In adults without pre-pandemic mhc, the series of Wilcoxon signed-rank tests indicated overall stronger effect sizes (r > .5), [43] on changes in emotions and worries than in participants with pre-pandemic mhc (see Tables 3 and A1 and A2 in online supplemental material). For both adults with and without pre-pandemic mhc, there was an increase in all means of emotions and worries from pre-pandemic (T0) to T2 (only partly significant) and a subsequent decrease (partly below the pre-pandemic level) at T3.

**Table 3:** Effect sizes *r* of Wilcoxon signed-rank on changes in emotions and worries about “be worried”, “enjoy activities” and “be happy or sad”

Item	Adults with pre-pandemic mhc (*n* = 32)						Adults without pre-pandemic mhc (*n* = 96)					
	pre-T1	T1-T2	T2-T3	T1-T3	pre-T2	pre-T3	pre-T1	T1-T2	T2-T3	T1-T3	pre-T2	pre-T3
Worried	0,42^a^	0,31^a^	**0,54**^b^	0,22^b^	0,61*^a^	0,22^a^	0,62*^a^	.23^a^	0,75*^b^	0,63*^b^	0,68*^a^	.06^b^
enjoy activities	0,41^a^	0,43^a^	0,69*^b^	0,40^b^	0,69*^a^	0,06^c^	0,61*^a^	.25^a^	0,62*^b^	.41*^b^	0,65*^a^	.23^a^
happy or sad	0,21^a^	**0,52**^a^	0,18^b^	0,33^a^	0,63*^a^	0,40^a^	.40*^a^	.49*^a^	0,53*^b^	.05^b^	0,67*^a^	.29*^a^

Effect sizes of r > .5 are displayed in bold

* Results are significant after FDR correction

^a^increase

^b^decrease

^c^no change

### Profile extraction

The three items of the *CRISIS* with the highest effect sizes of change in emotions and worries during the four time points (“be worried”, “enjoy activities” and “be happy or sad”, for each time point respectively) were selected for the LPA. Results of the LPA indicated that the best fitting model was a three-profile model with equal variances for each of the profiles, and covariances fixed to zero (meaning that the relationships between the variables are not estimated). This is a parsimonious model as less degrees of freedom than in the other models are used to explain the observations. The fit indices of the four models with two, three, and four profiles, respectively, are shown in Table A3 in online supplemental material.

**Fig. 2:**
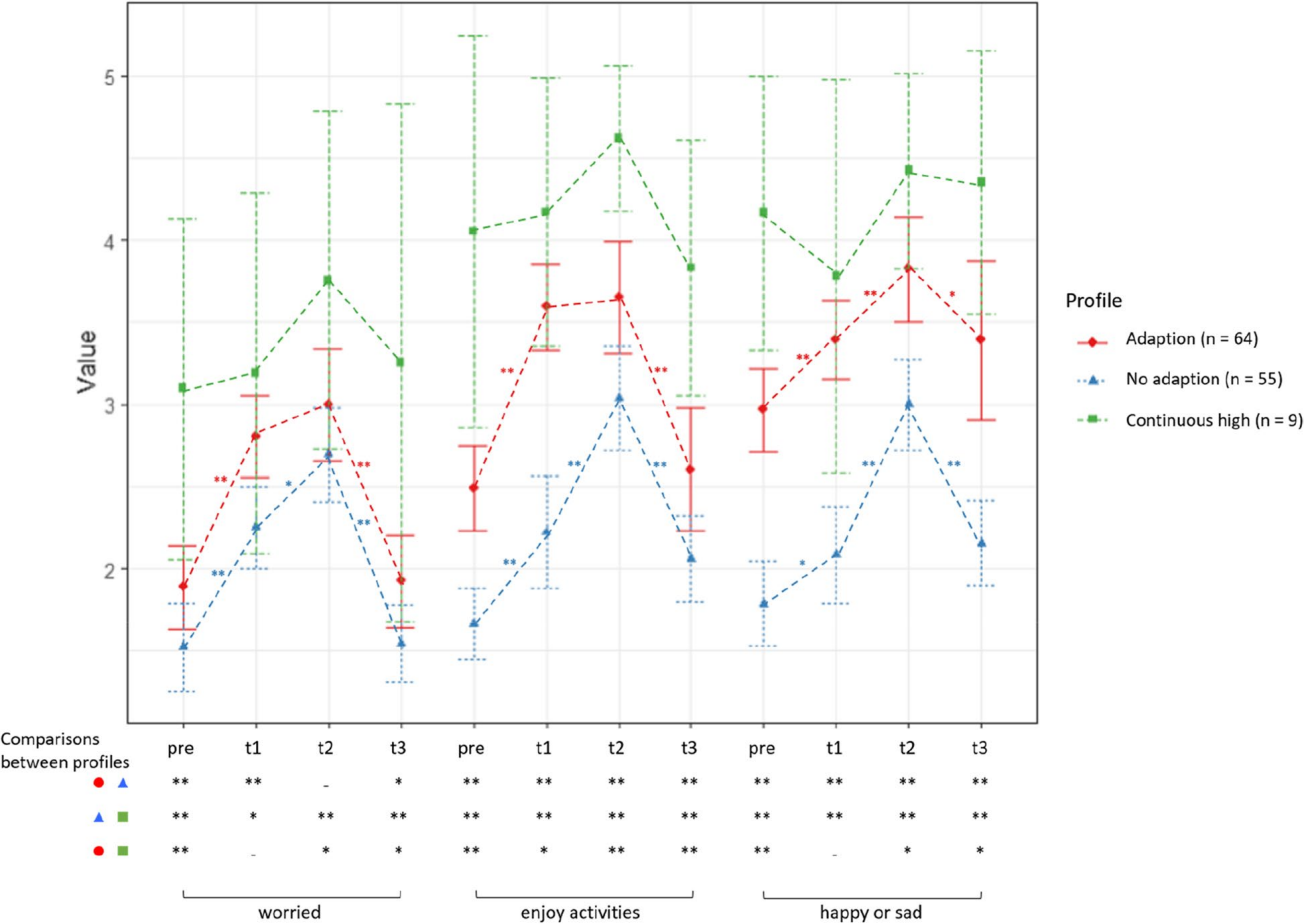
Mean values of the items “be worried”, “enjoy activities” and “be happy or sad” according to the three profiles during the 1.5-year study period. *Note.* Higher values indicate worsening of positive and negative emotions and worries. Comparisons between consecutive measures within a profile were run by Wilcoxon signed-rank tests. Comparisons between profiles at one time point were run by Mann-Whitney-U-tests. Pre = 3 months prior to the pandemic, t 1 = Spring 2020, t 2 = December 2020, t 3 = Summer/ autumn 2021 * *p* < .05; ** < .01; − not significant

The first of the three profiles (Fig. 2, red) contained 50% of the sample (*n* = 64) with a mean age of 39.95 (±10.08) years, 10.9% (*n* = 7) of them are male and 31.3% (*n* = 20) reported pre-pandemic mhc. The pre-pandemic values of the three included emotions and worries of profile 1 (worried *M*_*pre*_ = 1.91, enjoy activities, *M*_*pre*_ = 2.52, happy or sad *M*_*pre*_ = 2.98) ranges between the mean values of the other two profiles. There was a significant deterioration of be “worried” and “enjoy activities” from pre-pandemic to T1 (pre-T1: worried Z = − 5.39, *p* < .001, r = −.67; |enjoy activities Z = − 5.88, *p* < .001, r = −.74) but not from T1 to T2 (T1-T2: worried Z = − 1.51, *p* = .13, r = −.19; | enjoy activities Z = −.17, *p* = .87, r = −.02) and returns roughly to the pre-pandemic level at T3 (pre-T3: worried Z = −.15, *p* = .88, r = −.02; | enjoy activities Z = −.83, *p* = .41, r = − 10), while be “happy or sad” deteriorated from pre-pandemic to T1 (pre-T1: happy or sad Z = − 3.64, *p* < .001, r = −.45) and from T1 to T2 (T1-T2: happy or sad Z = − 3.11, *p* < .001, r = −.39) and remained above the pre-pandemic level at T3 (pre-T3: happy or sad Z = − 2.86, *p* < .001, r = −.36). The second of the three profiles (Fig. 2, blue) contained 43% of the sample (*n* = 55) with a mean age of 37.13 (±11.12) years, 20% (*n* = 11) of them were male and 9.1% (*n* = 5) reported pre-pandemic mhc. This profile has the lowest mean pre-pandemic values of the three included emotions and worries (worried *M*_*pre*_ = 1.53, enjoy activities, *M*_*pre*_ = 1.65, happy or sad *M*_*pre*_ = 1.80) and deteriorated from pre-pandemic to T1 (pre-T1: worried Z = − 4.29, *p* < .001, r = −.58; | enjoy activities Z = − 3.00, *p* < .001, r = −.40; | happy or sad Z = − 2.43, *p* = .02*, r = −.33) and from T1 to T2 (T1-T2: worried Z = − 2.29, *p* = .02*, r = −.31; | enjoy activities Z = − 3.82, *p* < .001, r = −.52; | happy or sad Z = − 4.51, *p* < .001, r = −.61) and remained above the pre-pandemic level at T3 for “enjoy activities” (pre-T3: worried Z = −.13, *p* = .90, r = −.02; | enjoy activities Z = − 2.78, *p* = .01, r = −.38; |happy or sad Z = − 2.33, *p* = .02*, r = −.31; *p*-values of .02* were no longer significant after FDR correction). The third of the three profiles (Fig. 2, green) contained 7% of the sample (*n* = 9) with a mean age of 34.78 (±10.11) years, none of them were male and 77.8% (*n* = 7) reported pre-pandemic mhc. Notably, this profile had the highest mean pre-pandemic values of the three included emotions and worries (worried *M*_*pre*_ = 3.11, enjoy activities, *M*_*pre*_ = 4.22, happy or sad *M*_*pre*_ = 4.22) but did not deteriorate from pre-pandemic to T1 (pre-T1: worried Z = −.06, *p* = .95, r − .02; | enjoy activities Z = .00, *p* = 1.00, r = .00; | happy or sad Z = -.63, *p* = .53, r = −.21) and deteriorated slightly but not significantly from T1 to T2 (T1-T2: worried Z = −.94, *p* = .35, r = −.31; | enjoy activities Z = − 1.41, *p* = .16, r = −.47; happy or sad Z = − 1.19, *p* = .24, r = −.40) and returned roughly to the pre-pandemic level at T3 (pre-T3: worried Z = −.34, *p* = .73, r = −.11; | enjoy activities Z = −.83, *p* = .41, r = −.28; | happy or sad Z = −.33, *p* = .74, r = −.11).

In sum, based on the three items “being worried”, “ enjoy activities” and “ be happy or sad”, we were able to identify three profiles of adults who show either an adaption to the pandemic (characterised by a strong worsening from T0 to T1 and no or less worsening from T1 to T2), no adaption (characterised by a worsening from T0 to T1 as well as T1 to T2) or a continuous high condition (characterised by significantly worse scores for emotions and worries even before the pandemic).

### Prediction of profile membership

We modelled the likelihood of predicting profile memberships with a multinomial logistic regression model. Due to the small sample size of profile three (*n* = 9) it was excluded for the regression model. Therefore, the dependent variable “profile membership” contained profiles one and two. The sample size of profiles one (*n* = 60) and two (*n* = 50) was reduced as a result of missing values of the predictor variables. Profile 2 was set as reference category. The model fit and odds ratios (OR) with their 95% confidence intervals are displayed in Table A4 in online supplemental material. Results of multinomial logistic regression analysis indicated that age, pre-pandemic mhc and perceived stress were significant predictors. In detail, the likelihood of an adaption rather than no adaption increases, if: participants were of older age compared to a younger age and participants had a higher perceived stress score compared to a lower perceived stress score. Participants with pre-pandemic mhc compared to participants without pre-pandemic mhc were more likely to show an adaption rather than no adaption (see Table A4 in online supplemental material.). The other predictors (sex, education, urban or rural living, minors in the same household, receiving social welfare, financial worries at T1) were not significant.

## Discussion

The present study provides insights into the different emotional responses of adults with and without pre-pandemic mhc to the COVID-19 related social restrictions and consequences during a 1.5-year period. Looking at the effect sizes of the comparison of two successive time points within the first nine months of the pandemic with severe social restrictions (T0 to T1 and T1 to T2), it seems as hypothesized - adults with pre-pandemic mhc experienced fewer negative changes in emotions and worries compared to adults without pre-pandemic mhc. A closer look at the period from pre-pandemic to the ninth month of pandemic (T0 to T2) reveals that adults with pre-pandemic mhc had a comparable degree of worsening of emotions and worries in this period as adults without pre-pandemic mhc. While adults without pre-pandemic mhc reported a strong worsening of emotions and worries at the beginning of the pandemic (T0 to T1) and a lower worsening during the course (T1 to T2), which reflect a normal pattern of stress reaction, adults with pre-pandemic mhc reported a constant worsening of emotions and worries from T0 to T1 to T2.

Speculatively, at the beginning of the pandemic, the social restrictions and their consequences might have been less burdensome for adults with pre-pandemic mhc. The reason might be that for adults with mhc, social contacts are often associated with anhedonia and fear, they often live socially withdrawn (e.g. [23]) and they might also have benefited from previously learned coping strategies [24], they experience easing of daily hassles [5] or having ongoing psychotherapy sessions [44]. Our findings of fewer negative changes in emotions and worries at the beginning of the pandemic in adults with pre-pandemic mhc compared to those without pre-pandemic mhc are in line with findings of another German study on adults with chronic mhc, with acute mhc and without mhc [45]. The authors compared perceived impairments in social participation from pre-pandemic as well as during and after the first lockdown. While adults with chronic mhc reported no additional impairment in social participation during the first lock-down, adults with acute mhc reported partly less impairment and adults without mhc reported partly more impairment during the first lockdown. Therefore, the results of the cited study and results of the present study suggest that adults with pre-pandemic mhc coped better with the social restrictions and consequences at the beginning of the pandemic. In addition, the results of our study suggest that on the long run the burden of COVID-19 related social restrictions and consequences in adults with pre-pandemic mhc increased and strategies used at the beginning of the pandemic could not stop the increasing load and exhaustion. On the other hand, adults without pre-pandemic mhc may have acquired strategies for dealing with the COVID-19 related social restrictions and consequences over time and thus prevented a further strong worsening of emotions and worries as happened at the beginning of the pandemic. Thus, the increase in emotions and worries from the pre-pandemic situation to the ninth month of the pandemic with social restrictions and consequences (T2) is comparable between adults with and without pre-pandemic mhc, but the pre-pandemic level and the dynamics of the increase differ. In summer/autumn 2021, after 1,5 years of pandemic situation, when regulations were considerably eased, emotions and worries subsided. Both adults with and without pre-pandemic mhc reached the pre-pandemic level in most items.

Independently of pre-pandemic mhc the results of the latent profile analysis revealed that some adults might have experienced fewer negative changes due to the COVID-19-related social restrictions and consequences or coped with them better than others (maybe during outdoor activities with family and friends in April/May, T1) at the beginning of the pandemic but struggled with them more and more after nine months of pandemic situation with social restrictions (December 2020, T2). They may have used more constructive coping strategies (e.g. positive reframing, acceptance, and humor) at the beginning of the pandemic, but struggled to maintain them over a long period such as nine months. A similar pattern was found in a representative German longitudinal study [46] demonstrating that life satisfaction decreased slightly in summer (during June 2020) and decreased even more in winter (during January and February 2021). A British longitudinal study also found such a pattern among mothers of young children (< 5 years) [47]. Their life satisfaction declined in spring and early summer 2020 (at the first lockdown) and decreased even more strongly in autumn/winter 2021 compared to pre-pandemic life satisfaction. The mothers of children aged 5–15 years were also found to experience the greatest decline in life satisfaction in autumn/winter 2021. However, this pattern is not evident among the fathers. In winter outdoor activities were difficult, which may have constrained previous coping strategies. On the other hand, our results revealed that there were also adults who experienced more negative changes due to the COVID-19-related social restrictions and consequences at the beginning of the pandemic but they seem to have gradually adapted coping strategies to the pandemic constraints. They may have changed their strategies from an emotion-divergent and avoidant coping style to a more constructive coping style. With the available data of our study, we identified increasing age and higher perceived stress as factors that seem to predict a more adaptive profile to the pandemic situation rather than an non-adaptive profile. At first glance, it might seem a bit irritating that higher age is associated with a more adaptive profile to the pandemic situation. This irritation is supported by another study, that showed that at the beginning of the pandemic the risk perception of being infected by COVID-19 decreases, while other worries about COVID-19 increase with older age [48]. The decreasing risk perception with older age is particularly astonishing against the background that, with increasing age probability of serious disease progression increases (i.e. [49]).

Notably, participants with pre-pandemic mhc compared to participants without pre-pandemic mhc were more likely to have an adaptive profile. Of the adults with pre-pandemic mhc (*n* = 32), 62.5% (*n* = 20) had an adaptive profile comparable to the change-pattern of adults without pre-pandemic mhc identified by the categorical approach. This suggests that the differences found in the categorical approach are driven by a small group of adults with pre-pandemic mhc and that a categorical approach with such a group comparison (with and without pre-pandemic mhc) seems to be too crude. Interestingly, other socio-demographic factors (e.g. sex, education, minors in the same household, social welfare) did not predict adaption to the pandemic situation. However, the findings of a study on changes in psychological vulnerability, resilience and social cohesion during COVID-19 give important indications regarding the present results [50]. In the cited study, results of latent chance score modelling revealed that high pre-pandemic levels of social cohesion were associated with stronger lockdown effects on mental health while high social cohesion during the lockdown and re-opening was associated with better mental health recovery. Furthermore, the authors found that the loss of social cohesion during the first lockdown was associated with a higher recovery of social cohesion after re-opening and concluded that the results “may speak to an initial shock response of individuals to an unpredicted collective stressor with a healthy recovery response afterward deploying the typically successful strategies of adaptive coping and seeking social support”. This might also apply to the adults with adaptive profile in the present study. In this context, coping flexibility may also have been relevant to how adults coped with the pandemic [51]. A further longitudinal community study over the first 1.5-years of the pandemic (start of the survey was during the first lock down) revealed that fears of negative social consequences (e.g., less contact to family and friends) corresponded to the intensity of social restrictions (in Germany) and reach the lowest level at the end of the 1.5-year study period at September 2021, which is comparable to the results of the present study from July to October 2021 (when social restrictions were considerably eased or stopped and contact to family and friends was easier again.), with means of emotions and worries returning to the initial pre-pandemic levels [52].

The present study has limitations that need to be considered. The main limitation is that emotions and worries for two time points (pre-pandemic and T2) were measured retrospectively and, therefore, the recall bias might have affected our results. However, the CRISIS questionnaire in US and UK population samples demonstrated construct validity and a high reproducibility of subtypes of life changes from before the pandemic to during the pandemic [34]. The longest time lag in the present study was for the retrospective survey for the hard lockdown at Christmas time in December 2020 (around 6 months), for which participants rated emotions and worries worst of all four time points. This is plausible, as many families traditionally get together at Christmas time, even those who rarely see each other during the rest of the year, and the hard lockdown restricted this considerably. It should be noted that events that are perceived as negative or episodes with a negative valence are remembered more accurately even over a longer period of time than positive ones [53–55], while positive emotions can retrospectively be overestimated [53]. We therefore assume that the bias for T2 is low and that this is actually the most negatively experienced time point in the present study. A further limitation is that the data of the present study represent a sample of families from a small region in Germany and is, therefore, not a populational based sample representative for the general population in Germany or Europe. In addition, in the group without pre-pandemic mhc females are overrepresented and in the group with pre-pandemic mhc all participants are female. Even if mental disorders are more frequent in females than in males [56], the lack of male participants in the group with mhc again shows that the present sample is a selective sample that does not allow conclusions to be drawn about the general population. One explanation for the overrepresentation of female participants could be that for former or current minor patients or study participants, mothers are more often our contact person than fathers. As a result, mothers were contacted more frequently than fathers when parents and children were initially asked to participate in the study. However, the frequency of mhc in the present sample (25%) corresponds to the prevalence of mental disorders in Europe (lifetime prevalence of any mental disorder 25% [56]). A further limitation arises from the small sample size. The sample size of the present study is quite small for complex analysis such as a LPA. The continuous high profile with only 9 participants is not suitable for interpretation and any general conclusions. In terms of the two other profiles (adaption and no adaption), a recent study demonstrated that stable LPA models can be identified even in small sample sizes (e.g. *n* = 120 [57]). Also, due to the small sample size of adults with pre-pandemic mhc, the data were not suitable to provide information on changes of emotions and worries in different mental disorders. Furthermore, there are some important co-variates that may have had an effect on the emotions and worries of the participants with mhc (e.g. medication, current therapy) which were not captured in the present study. For studies on mental disorders, the challenge is to reach a sufficient sample size for such analyses with detailed information about the participants. Finally, the low response rate should be noted. Of 352 people who were asked to complete the online questionnaires at T2 and T3 again, only 128 (36.4%) reported on all four time points. This high dropout rate might have biased the present results as worse physical and cognitive functioning is associated with higher study dropout [58]. Therefore, mainly people with physical and mental impairments may have dropped out of the present study. In sum, the present study has a number of limitations and does not allow conclusions to be drawn about the general population. In a larger and representative sample, however, we would expect that the identified profiles will be replicated with additional profiles that might emerge and a more specific prediction of the profiles.

## Conclusion

In terms of the impact of the COVID-19 related social restrictions and consequences on emotions and worries, this study on families of current and former patients or study participants of a child and adolescent psychiatry provides further evidence that some adults coped well at the beginning of the pandemic but became more stressed over time, while others struggled more with social restrictions and consequences at the beginning of the pandemic but adapted their coping strategies to the constraints of the pandemic over time. Adults with pre-pandemic mhc may have benefited from previously learned coping strategies and social restrictions may have less burdensome for them. Thus, the results of the present study suggests that adults (both with and without pre-pandemic mhc) coped the crisis with different strategies and that most of them returned to their initial, pre-pandemic levels when social restrictions were considerably eased or stopped. So far, research on coping strategies has mainly focused on individual stressors such as disturbing life events or mental health conditions such as anxiety or depression. Such coping strategies often use social contacts as resources. Strategies to cope with severe social restrictions and the dynamics of collective stressors rather than individual stressors have been less studied so far. Future research should synthesise the findings from the COVID-19 pandemic and further explore the dynamics of collective stressors under conditions of social restriction, considering cultural differences at the regional level.

### Supplementary Information

**Additional file 1.**

## Data Availability

The datasets used and/or analysed during the current study are available from the corresponding author on reasonable request.

## Abbreviations

COVID-19: Corona virus disease 2019
mhc: Mental health condition
SARS-CoV-1: Severe acute respiratory syndrome corona virus 1
MERS: Middle east respiratory syndrome
CRISIS: CoRonavIruSHealth impact survey
PSS-10: Perceived stress scale
FDR: False discovery rate
LPA: Latent profile analysis
BIC: Bayesian information criterion
AIC: Akaike’s information criterion
AWE: Approximate weight of evidence
CLC: Classification likelihood criterion
KIC: Kullback information criterion
