# Supplementary material for: Emotions and worries during 1.5 years of the COVID-19 pandemic - how adults with and without mental health conditions coped with the crisis
Source: BMC Psychiatry. 2024 Feb 9;24:114. doi: 10.1186/s12888-024-05573-x (PMC10858480; doi:10.1186/s12888-024-05573-x)
Supplement: Supplementary file 1 — Additional file 1. [file 12888_2024_5573_MOESM1_ESM.pdf]

Online supplement material

Emotions and Worries of the CoRonavIruS Health Impact Survey (CRISIS)

How worried were you generally?

Not worried at all - Slightly worried - Moderately worried - Very worried - Extremely worried

How happy versus sad were you?

Very happy/cheerful - Moderately happy/cheerful - Neutral - Moderately sad/depressed/unhappy - Very sad/depressed/unhappy

How much did you enjoy your usual activities?

A lot - Very much - Moderately - Slightly - Not at all

How relaxed versus anxious were you?

Very relaxed/calm - Moderately relaxed/calm - Neutral - Moderately nervous/anxious - Very nervous/anxious

How fidgety or restless were you?

Not restless at all - Slightly restless - Moderately restless - Very restless - Extremely restless

How fatigued or tired were you?

Not fatigued or tired at all - Slightly fatigued or tired - Moderately fatigued or tired - Very fatigued or tired - Extremely fatigued or tired

How well were you able to concentrate or focus?

Very focused/attentive - Moderately focused/attentive - Neutral - Moderately unfocused/distracted - Very unfocused/distracted

How irritable or easily angered were you?

Not irritable or easily angered at all - Slightly irritable or easily angered - Moderately irritable or easily angered - Very irritable or easily angered - Extremely irritable or easily angered

How lonely were you?

Not lonely at all - Slightly lonely - Moderately lonely - Very lonely - Extremely lonely

To what extent have you had negative thoughts?

Not at all - Rarely - Occasionally - Often - A lot of the time

Situation in Germany during data collection

Spring 2020 (retrospective pre-pandemic inquiry and T1 data collection)

The first lockdown was implemented on March 20th, supposedly lasting for two weeks but was prolonged for another two weeks until April 20th. During this time only shops of everyday needs were allowed to open, and the first curfews were imposed, restricting social contacts and scope of movement. It is important to mention that most employees had to work from home while simultaneously child daycare-centers (kindergarten, schools etc.) were being closed. Aggravatingly, staying outside was forbidden except for taking a walk or a jog within a specified radius (playgrounds were closed).

December 2020 (retrospective inquiry T2)

During the summer period the amount of (daily) cases reduced significantly, hence social restrictions were eased. Social life went almost back to normal. In autumn incidences started rising again, resulting in a new partial lockdown at the beginning of November (02/11/2020) and a “hard lockdown” in December with even more social restrictions being imposed in order to prevent infection spread. All cultural and social facilities (e.g., restaurants, bars, clubs) were being closed, as well as shopping facilities that did not concern daily needs (e.g., clothing stores). Social gatherings were being restricted – per household only one additional person was allowed (a total of five persons was not to be exceeded). A curfew prohibited leaving home between 10 pm and 6 am (exceptions: e.g., professional obligations). Additionally, schools and kindergarten were closed again. Schools first announced early Christmas holidays and then changed to a model where online and real-life classes rotated. The second lockdown additionally took place during a crucial point of time, due to the importance of the Christmas period in Germany which is normally a time of frequent social gatherings and events and were restricted vigorously over the Christmas holidays. That is important to mention as social rituals and events are shown to increase wellbeing and life satisfaction [1]. On the other hand, Christmas often is associated with higher levels of loneliness and reduced emotional wellbeing especially in those suffering from mental health illnesses [2, 3].

Summer/ autumn 2021 (T3 data collection)

In contrast, the situation during summer and autumn 2021 (July 16th –October 25th) was less restricted. The vaccination campaign had made tremendous progress over the past couple of months, resulting in 67% of the German population that had been already fully vaccinated (October 22nd). In July, August and September 7-day incidences in Saxony had been rather small (July: < 10; August: < 20; September: up until 50) for a few weeks, resulting in a considerable ease of regulations and a vast opening of social facilities (e.g., theatres, cinemas, clubs etc.) for those fully vaccinated, negatively tested or with officially confirmed recuperation and previously state-mandatory remote work was not state regulated any longer. However, it is noteworthy that throughout October, case-numbers and the number of severe cases had already started to rise again – possibly increasing worries about new regulations or even a lockdown especially towards end of October or beginning of November.

Scales of socio-demographic measures

Age, Sex (male, female, divers), Education (no graduation/lower secondary/middle secondary/upper secondary/post-secondary non-tertiary/ Bachelor, Master, Doctoral or equivalent), Urban (or rural) living (large city, suburb of a large city, medium-sized city, small town, rural area), Minors living in the same household (yes/no), Pre-pandemic mhc (yes/no), Get social welfare (yes/no), Financial worries (not at all, somewhat, moderate, much, extreme).

Table A1. Wilcoxon-signed rank test comparing emotions and worries of adults with pre-pandemic mhc from pre-pandemic values (2020) until summer/autumn 2021

Item	**Adults with mhc (n = 32)**																	
	pre vs. T1			T1 vs. T2			T2 vs. T3			T1 vs. T3			pre vs. T2			pre vs. T3		
	Z	p	r	Z	p	r	Z	p	r	Z	p	r	Z	p	r	Z	p	r
Worried	-2.40	.016	-0,42	-1.78	.075	-0,31	-3.07	.002	-0,54	-1.25	.212	-0,22	**-3.43**	**<.001**	**-0,61**	-1.24	.215	-0,22
enjoy activities	-2.30	.022	-0,41	-2.42	.016	-0,43	**-3.90**	**<.001**	**-0,69**	-2.26	.024	-0,40	**-3.90**	**<.001**	**-0,69**	-.33	.739	-0,06
Concentrated	-1.00	.315	-0,18	-1.44	.151	-0,25	-.75	.454	-0,13	-.50	.614	-0,09	-2.12	.034	-0,37	-1.49	.137	-0,26
Lonely	-.06	.954	-0,01	-2.75	.006	-0,49	-2.23	.026	-0,39	-.96	.335	-0,17	-2.59	.010	-0,46	-.63	.530	-0,11
negative thoughts	-1.06	.287	-0,19	-2.00	.045	-0,35	-.56	.575	-0,10	-1.29	.196	-0,23	-1.21	.225	-0,21	-.82	.413	-0,14
happy or sad	-1.18	.240	-0,21	-2.96	.003	-0,52	-1.02	.307	-0,18	-1.89	.058	-0,33	**-3.56**	**<.001**	**-0,63**	-2.27	.023	-0,40
relaxed or anxious	-1.36	.175	-0,24	-1.65	.099	-0,29	-1.37	.172	-0,24	-.43	.668	-0,08	-2.14	.032	-0,38	-.48	.628	-0,08
fidgety or restless	-.08	.936	-0,01	-1.78	.075	-0,31	-1.56	.119	-0,28	-.25	.805	-0,04	-1.85	.064	-0,33	-.52	.605	-0,09
fatigued or tired	-.23	.818	-0,04	-2.64	.008	-0,47	-.94	.346	-0,17	-1.54	.124	-0,27	-2.20	.028	-0,39	-1.21	.226	-0,21
irritable or easy angered	-1.15	.250	-0,20	-1.45	.148	-0,26	-.22	.829	-0,04	-.90	.366	-0,16	-1.65	.099	-0,29	-2.21	.027	-0,39

Note. Results displayed in bold are significant after FDR correction.

Table A2. Wilcoxon-signed rank test comparing emotions and worries of adults without pre-pandemic mhc from pre-pandemic values (2020) until summer/autumn 2021

Item	**Adults without mhc (n = 96)**																	
	pre vs. T1			T1 vs. T2			T2 vs. T3			T1 vs. T3			pre vs. T2			pre vs. T3		
	Z	p	r	Z	p	r	Z	p	r	Z	P	r	Z	p	r	Z	p	r
Worried	**-6.11**	**<.001**	**-0,62**	-2.22	.027	-0,23	**-7.31**	**<.001**	**-0,75**	**-6.16**	**<.001**	**-0,63**	**-6.65**	**<.001**	**-0,68**	-.59	.559	-0,06
enjoy activities	**-5.94**	**<.001**	**-0,61**	-2.47	.014	-0,25	**-6.10**	**<.001**	**-0,62**	**-3.97**	**<.001**	**-0,41**	**-6.37**	**<.001**	**-0,65**	-2.30	.022	-0,23
Concentrated	**-2.83**	**.005**	**-0,29**	-.76	.449	-0,08	-1.98	.048	-0,20	-1.33	.184	-0,14	**-3.42**	**<.001**	**-0,35**	-1.77	.077	-0,18
Lonely	**-4.09**	**<.001**	**-0,42**	-.40	.687	-0,04	-2.66	.008	-0,27	-2.32	.021	-0,24	**-3.27**	**<.001**	**-0,33**	-.86	.391	-0,09
negative thoughts	-.041	.967	0,00	-.95	.341	-0,10	**-2.93**	**.003**	**-0,30**	-1.89	.058	-0,19	-.92	.358	-0,09	-1.75	.079	-0,18
happy or sad	**-3.96**	**<.001**	**-0,40**	**-4.82**	**<.001**	**-0,49**	**-5.24**	**<.001**	**-0,53**	-.50	.621	-0,05	**-6.52**	**<.001**	**-0,67**	**-2.80**	**.005**	**-0,29**
relaxed or anxious	**-3.39**	**<.001**	**-0,35**	-2.57	.010	-0,26	**-5.17**	**<.001**	**-0,53**	**-3.07**	**.002**	**-0,31**	**-4.60**	**<.001**	**-0,47**	-.08	.940	-0,01
fidgety or restless	-2.62	.009	-0,27	-1.44	.151	-0,15	**-3.05**	**.002**	**-0,31**	**-3.64**	**<.001**	**-0,37**	-.47	.638	-0,05	-1.93	.053	-0,20
fatigued or tired	-.34	.733	-0,03	**-3.27**	**.001**	**-0,33**	**-3.64**	**<.001**	**-0,37**	-.74	.461	-0,08	**-3.01**	**.003**	**-0,31**	-1.20	.232	-0,12
irritable or easy angered	**-3.23**	**.001**	**-0,33**	-1.71	.086	-0,17	**-3.71**	**<.001**	**-0,38**	-2.01	.044	-0,21	**-4.04**	**<.001**	**-0,41**	-.81	.419	-0,08

Note. Results displayed in bold are significant after FDR correction.

Table A3. Latent profile analysis fit indices.

Model	Profiles	BIC	AIC	AWE	CLC	KIC	Entropy
Equal variances, covariances fixed to 0							
	2	4349.45	4243.92	4638.28	4171.62	4283.92	.85
	3	4331.78	4189.18	4722.62	4090.95	4242.18	.88
	4	4351.84	4172.16	4844.85	4047.83	4238.16	.83
Varying variances, covariances fixed to 0							
	2	4379.08	4239.33	4762.11	4143.06	4291.33	.86
	3	4399.50	4188.45	4978.82	4042.19	4265.45	.87
	4	4483.89	4201.54	5259.43	4005.36	4303.54	.91
Equal variances, equal covariances							
	2	4344.18	4050.42	5151.26	3846.09	4156.42	.84
	3	4387.22	4056.38	5296.38	3826.05	4175.38	.83
	4	4395.66	4027.75	5406.79	3771.53	4159.75	.89
Varying variances, varying covariances							
	2	4526.20	4009.99	5945.49	3649.92	4193.99	.97
	3	4851.12	4075.37	6984.95	3533.29	4350.37	.96
	4	5126.19	4090.90	7974.54	3366.85	4456.90	.97

[1] best fitting model. BIC = Bayesian information criterion, AIC = Akaike information criterion, AWE = approximate weight of evidence, CLC = classification likelihood criterion, KIC = Kullback information criterion. Lower values indicate a better fitting model. Entropy values ≥0.80 correspond to an acceptable degree of separation between classes

Table A4. Model fit and odds ratio (OR) with 95%t confidence intervals (CI) of multinomial logistic regression

Effect	Model fitting criteria	Likelihood ratio test			95% CI of odds ratio		
	-2 log likelihood of reduced model	X^2	p	OR	Lower bound	Upper bound	p
**Likelihood ratio tests**							
age	97.68	5.67	.02	1.1	1.01	1.20	.03
sex	92.02	.01	.94	1.06	.23	5.02	.94
education	92.14	.12	.73	1.05	.79	1.41	.73
urban or rural living	92.77	.75	.39	1.20	.79	.1.81	.39
minors in the same household	93.65	1.63	.20	3.63	.48	27.56	.21
no pre-pandemic metal health condition	97.08	5.06	.03	.21	.05	.87	.03
receive social welfare	92.16	.15	.70	.60	.04	8.50	.70
financial worries at T1	92.77	.74	.39	1.41	.65	3.08	.39
perceived stress at T1	133.52	41.50	<.01	1.17	1.10	1.25	<.01

				Pearson			Nagelgerke R^2
	-2 log likelihood			X^2	df	p	
**Final model fitting statistics**							
	92.02	59.57	<.001	102.07	100	.42	.56

Note. Profile 2 was set as reference category

References

1. Páez D, Rimé B, Basabe N, Wlodarczyk A, Zumeta L. Psychosocial effects of perceived emotional synchrony in collective gatherings. Journal of Personality and Social Psychology. 2015;108:711–29.

2. Sansone RA, Sansone LA. The Christmas Effect on Psychopathology. Innov Clin Neurosci. 2011;8:10–3.

3. Velamoor VR, Cernovsky ZZ, Voruganti LP. Psychiatric Emergency Rates during the Christmas Season in the Years 1991 to 1997. Psychol Rep. 1999;85:403–4.
